# Impact of age on the toxicity of immune checkpoint inhibition

**DOI:** 10.1136/jitc-2020-000871

**Published:** 2020-10-08

**Authors:** Amit Samani, Shuai Zhang, Laura Spiers, Ali Abdulnabi Suwaidan, Sophie Merrick, Zayd Tippu, Miranda Payne, Guy Faust, Sophie Papa, Paul Fields, Mieke Van Hemelrijck, Debra H Josephs

**Affiliations:** 1 Department of Surgery and Cancer, Imperial College London, London, London, UK; 2 School of Cancer and Pharmaceutical Sciences, King's College London, London, UK; 3 Department of Oncology, University Hospitals of Leicester NHS Trust, Leicester, Leicester, UK; 4 Guy's Cancer Centre, Guy's and St. Thomas' NHS Foundation Trust, London, London, UK; 5 Department of Oncology, University of Oxford & Oxford Cancer Centre, Oxford University Hospitals NHS Foundation Trust, Oxford, Oxfordshire, UK; 6 Department of Haematology, Guy's and St. Thomas' NHS Foundation Trust, London, London, UK; 7 Translational Oncology and Urology Research (TOUR), School of Cancer and Pharmaceutical Sciences, King's College London, Guy's Hospital, London, UK

**Keywords:** immunotherapy, programmed cell death 1 receptor, self tolerance

## Abstract

Indications for immune checkpoint inhibitor therapy are increasing. As the population ages, many patients receiving such drugs will be older adults. Such patients are under-represented in clinical trials, and therefore the safety of immune checkpoint inhibitors in this population has not been adequately assessed. A retrospective multicenter analysis of toxicities was performed in patients with advanced or metastatic solid cancers receiving anti-programmed cell death protein 1 (anti-PD-1) and/or anti-CTLA4 antibodies across three age cohorts (<65 years, 65–74 years and ≥75 years) using univariable and multivariable analyzes. Eligible patients (n=448) were divided into age cohorts: <65 years (n=185), 65–74 years (n=154) and ≥75 years (n=109). Fewer patients in the oldest cohort (7.3%) received an anti-CTLA4 antibody containing regimen compared with the younger cohorts (21.1% and 17.5%). There was no significant difference overall in all grade or ≥G3 toxicities between age cohorts. Significantly fewer patients in the older (65–74 years and ≥75 years) age cohorts discontinued treatment because of toxicity (10.1% and 7.4%) compared with in the <65 years cohort (20.5%; p=0.006). Using logistic regression, only treatment type (ipilimumab containing) was significantly associated with all grade toxicity. However, there was a significantly lower incidence of all-grade endocrine toxicity in the oldest cohort (11.0%) compared with the youngest cohort (22.7%, p=0.02; OR 0.43, 95% CI 0.21 to 0.87), while all-grade dermatological toxicity showed the reverse trend (28.4% vs 18.9%; OR 1.85, 95% CI 1.04 to 3.30). Results were corroborated in the sensitivity analysis using only data from patients who received PD-1 inhibitor monotherapy. This multicenter, real-world cohort demonstrates that immune checkpoint inhibitor therapy is safe and well tolerated regardless of age, with no appreciable increase in adverse events in older adult patients.

## Introduction

Cancer incidence increases with age. There is an 11-fold higher incidence in patients over 65 years compared with younger patients.^1^ As life expectancy increases, the number of over 65s worldwide is predicted to rise by 78% and in Europe and North America, the proportion of over 65s could reach 25% by 2050.^2^ Despite this, the older population tends to be under-represented in oncology clinical trials. A recent study pooling 25 European Organisation for Research and Treatment of Cancer adult oncology randomized controlled trials (RCTs) showed that of more than 6000 patients, only 9% were aged 70 or older.^3^

Immune checkpoint inhibitors (ICIs) for patients with cancer are generally well tolerated, especially as monotherapy, when compared with traditional chemotherapy. However, ICIs are associated with a spectrum of adverse events, known as immune-related adverse events, many of which phenotypically resemble endogenous autoimmune or autoinflammatory conditions.^4^ Aging affects both innate and adaptive immune function and the incidence/pattern of autoimmune conditions. For example, there is an increase in the basal inflammatory process with age, and an elevated production of autoantibodies, with a concomitant increase in certain autoimmune pathologies.^5 6^ Conversely, the process of age-related immunosenescence may dampen intrinsic immune processes, which may in-turn affect the efficacy of immunotherapies. For example, as age increases there is a decrease in the production of naïve T cells, a decline in hematopoietic stem cell generation of T cell precursors, and a more restricted T cell receptor repertoire.^7 8^ T cell signaling through the T cell receptor also decreases.^9^ Finally, aged T cells display an increased level of inhibitory immune checkpoints such as PD-1, Lag-3 and Tim-3.^9 10^ The multiple factors described above may cause the pattern of ICI induced toxicities to be different in older patients compared with younger cohorts. However, only a minority of patients recruited to the registration trials of ICIs were aged above 75, and therefore, toxicity estimates in this cohort are unreliable.^11–16^

While the theoretical effect of age on the toxicity of immunotherapy is uncertain, the clinical evidence for safety of ICIs in the older patient population is also conflicting. Some studies have shown little difference with age, while others suggest that advancing age confers a protective effect against development of toxicities.^17–23^ However, to date, the post-RCT studies of this topic have been single-center studies with fewer than 250 patients. Here, we conduct a large, multicenter study of the toxicity of immune checkpoint inhibition across various age cohorts, in a real-world setting.

## Methods

### Data source

Data were collected retrospectively from electronic patient records and inpatient notes from three university hospitals within the UK: Guy’s and St Thomas’ National Health Service (NHS) Foundation Trust (London), Oxford University Hospital NHS Foundation Trust (Oxford) and University Hospitals of Leicester NHS Trust (Leicester).

### Study population

Included patients were aged **≥**18 years and had received at least one dose of anti-PD-1 and/or anti-CTLA4 antibody between October 2014 and June 2017 for melanoma, non-small-cell lung cancer or renal cell carcinoma. Pembrolizumab was administered at a dose of either 3 mg/kg or a 200 mg flat dose, 3 weekly. Nivolumab monotherapy was given as 3 mg/kg, 2 weekly. When given in combination, nivolumab and ipilimumab were given at 1 mg/kg and 3 mg/kg 3 weekly for four doses, respectively. Ipilimumab as monotherapy was administered as 3 mg/kg 3 weekly.

Baseline data were collected on age, gender, primary tumor site and treatment start date. Safety data included the nature, date and grade of toxicity (G, scored according to the Common Terminology Criteria for Adverse Events V.4.03), the number of immunotherapy cycles received and reason for discontinuation. Toxicities/adverse events were classified according to organ systems affected, that is, dermatological, gastrointestinal (GI), endocrine, hepatic, rheumatological and other toxicities. For patients who received ≥2 lines of immunotherapy with different agents, toxicity was only counted if it occurred prior to the date of commencement of the second line. Data on treatment modalities used for toxicities ≥grade 3 were also collected.

Patients were categorized into three age groups, according to age at treatment initiation: <65 years, 65–74 years and ≥75 years. These groups were selected based on the paucity of currently available data for patients ≥75 years, and bearing in mind current and predicted future patient demographics.

### Statistical analysis

Statistical analyzes were performed using R version 4.0.0, SPSS (V.25, IBM) and GraphPad Prism (V.8.0, Graphpad Software, San Diego, USA). The start date of the study was date of first treatment and last date of the study was date of death or 1 March 2018, whichever occurred first.

Descriptive analyzes were used to summarize study sample characteristics and toxicity data. The proportion of toxicities was compared across age categories using the Pearson X^2^, or X^2^ test for trend. Univariable analyzes were performed to examine the individual effect of age category, primary tumor site or treatment type on toxicity.

To take into account the effect of multiple factors on toxicity, multivariable analyzes were also performed. Age category, primary tumor site and treatment type were used as independent variables and toxicity was modeled as the dependent variable. Logistic regression was used to generate odds ratios for the independent variables.

A sensitivity analysis was implemented by excluding those who were treated with ipilimumab-containing regimens. This was to determine whether those receiving ipilimumab-containing regimens were having a disproportionate impact on the results, since such regimens have an established greater toxicity profile than PD-1 inhibitor monotherapy (pembrolizumab and nivolumab).^24^ This cohort was analyzed using the same descriptive statistics and univariable analyzes as described above.

## Results

Baseline demographics are shown in table 1. The 448 eligible patients were divided into three age cohorts: <65 years (n=185), 65–74 years (n=154) and ≥75 years (n=109). The median age of patients in the ≥75 years cohort was 79 (range 75–96). Fewer patients in the oldest cohort received an ipilimumab containing regimen (7.3%) compared with the younger cohorts (17.5% in 65–74 years; 21.1% in <65 years).

**Table 1:** Baseline and toxicity characteristics overall and by age

	Overall N (%)		<65 years N (%)		65–74 years N (%)		≥75 years N (%)		X^2^	
**No of patients (%**)	448 (100)		185 (41.3)		154 (34.4)		109 (24.3)			
**Median age (range**)	67 (21–96)		55 (21–64)		70 (65–74)		79 (75–96)			
**Primary site (%**)										
Melanoma	258 (57.6)		109 (58.9)		74 (48.1)		75 (68.8)			
Lung	116 (25.9)		36 (19.5)		58 (37.7)		22 (20.2)			
Renal	74 (16.5)		40 (21.6)		22 (14.3)		12 (11.0)			
**First-line treatment (%**)										
Pembrolizumab	287 (64.1)		99 (53.5)		102 (66.2)		86 (78.9)			
Nivolumab	87 (19.4)		47 (25.4)		25 (16.2)		15 (13.8)			
Ipilimumab+nivolumab	54 (12.1)		28 (15.1)		23 (14.9)		3 (2.8)			
Ipilimumab	20 (4.5)		11 (6.0)		4 (2.6)		5 (4.6)			
**Median no of cycles** (**range**)	6 (1–60)		5 (1–60)		7 (1–49)		7 (1–33)			
	**All grade**	**≥G3**	**All grade**	**≥G3**	**All grade**	**≥G3**	**All grade**	**≥G3**	**All grade**	**≥G3**
**All toxicity**	270 (60.3)	72 (16.1)	111 (60.0)	35 (18.9)	97 (63.0)	25 (16.2)	62 (56.9)	12 (11.0)	NS	NS
**Adverse event**										
Dermatitis	100 (22.3)	4 (0.9)	35 (18.9)	0 (0.0)	34 (22.1)	2 (1.3)	31 (28.4)	2 (1.8)	NS	NS
Lower GI	88 (19.6)	27 (6.0)	37 (20.0)	15 (8.1)	32 (20.8)	7 (4.6)	19 (17.4)	5 (4.6)	NS	NS
Endocrine	88 (19.6)	11 (2.5)	42 (22.7)	4 (2.2)	34 (22.1)	5 (3.3)	12 (11.0)	2 (1.8)	**0.02**	NS
Hepatitis	56 (12.5)	16 (3.6)	30 (16.2)	6 (3.2)	16 (10.4)	6 (3.9)	10 (9.2)	4 (3.7)	NS	NS
Rheumatological	25 (5.6)	4 (0.9)	7 (3.8)	2 (1.1)	10 (6.5)	2 (1.3)	8 (7.3)	0 (0.0)	NS	NA
Other	77 (17.2)	18 (4.0)	32 (17.3)	9 (4.9)	31 (20.1)	7 (4.6)	14 (12.8)	2 (1.8)	NS	NS
	**Overall N (%**) (**n=302**)		**<65 years N (%**) (**n=112**)		**65–74 years N (%) (n=109**)		**≥75 years N (%**) (**n=81**)		**X^2^**	
**Discontinuation due to toxicity**	40 (13.2)		23 (20.5)		11 (10.1)		6 (7.4)		**0.006**	

A total of 448 patients overall for which toxicity data are known, 302 patients for which continuation/discontinuation data are known.

GI, gastrointestinal; NA, Insufficient patient numbers for analysis; NS, not significant.

Across all age cohorts 270 patients (60.3%) experienced toxicity of any grade, with 72 (16.1%) developing at least one ≥G3 event (table 1). The most common all-grade toxicity was dermatitis (22.3%), and the most common ≥G3 toxicity was lower GI, affecting 6.0% of patients. Of patients for whom discontinuation data were available (n=302), 40 patients (13.2%) discontinued treatment because of toxicity, after a median of 3 cycles (range 1–43) (table 1).

The proportion of patients with overall all-grade and ≥G3 toxicity was similar across age cohorts (table 1) with a trend toward lower overall all-grade and ≥G3 toxicity in the ≥75 years group (56.9% and 11.0%), compared with the <65 years and 65–74 years cohorts (60.0% and 18.9%; 63.0% and 16.2%, respectively). In parallel with this, a significantly smaller proportion of patients in the 65–74 years and ≥75 years age cohorts discontinued treatment because of toxicity (10.1% and 7.4%) compared with those in the <65 years cohort (20.5%; p=0.006). There was a statistically significant difference in all grade endocrine toxicity, which was highest in the younger two cohorts (22.7% and 22.1%) relative to the oldest cohort (11.0%; p=0.02). Conversely, there was a non-significant trend toward higher rates of dermatological toxicity in the oldest cohort, with 28.4% of patients affected vs 18.9% and 22.1% in the younger cohorts (<65 years and 65–74 years, respectively, table 1).

Next, logistic regression was used to assess the relative influence of age category, primary tumor site and treatment type (both in univariable and multivariable analyzes) on determining the risk of toxicity in patients receiving ICIs. Using logistic regression, only treatment type was significantly associated with all grade toxicity, with the risk of toxicity significantly greater in those patients treated with an ipilimumab-containing regimen (OR 5.24; 95% CI 1.48 to 18.50 for ipilimumab/nivolumab combination relative to PD-1 inhibitor monotherapy) (table 2).

**Table 2:** Logistic regression analysis of risk of toxicity in patients receiving immune checkpoint inhibitors

	All-grade toxicity		≥G3 toxicity	
	Univariate	Multivariable	Univariate	Multivariable
**Age category**				
<65 years	1.00 (ref)	1.00 (ref)	1.00 (ref)	1.00 (ref)
65–74 years	1.14 (0.73 to 1.76)	1.27 (0.79 to 2.03)	0.825 (0.47 to 1.45)	0.96 (0.51 to 1.81)
≥75 years	0.88 (0.54 to 1.42)	1.15 (0.70 to 1.91)	0.53 (0.26 to 1.07)	0.73 (0.33 to 1.61)
**Primary site**				
Melanoma	1.00 (ref)	1.00 (ref)	1.00 (ref)	1.00 (ref)
NSCLC	0.79 (0.51 to 1.24)	0.95 (0.59 to 1.53)	0.50 (0.26 to 0.96)	0.79 (0.38 to 1.62)
RCC	1.02 (0.60 to 1.74)	1.50 (0.86 to 2.61)	0.41 (0.18 to 0.95)	0.90 (0.36 to 2.22)
**Treatment type**				
PD-1 inhibitor	1.00 (ref)	1.00 (ref)	1.00 (ref)	1.00 (ref)
Ipilimumab+nivolumab	4.72 (1.36 to 16.39)	5.24 (1.48 to 18.50)	17.94 (6.71 to 47.91)	16.41 (5.88 to 45.84)
Ipilimumab	8.17 (3.18 to 20.96)	8.93 (3.43 to 23.29)	7.73 (4.08 to 14.65)	7.02 (3.53 to 13.96)

OR with 95% CI for toxicity using logistic regression.

NSCLC, non-small cell lung cancer; RCC, renal cell carcinoma.

Based on the results of the univariate analyzes, we also specifically analyzed dermatological and endocrine toxicities using multivariate logistic regression (table 3). The OR for endocrine toxicity was 0.43 (95% CI 0.21 to 0.87) for those aged ≥75 as compared with those <65. Conversely, the OR for dermatological toxicity was 1.85 (95% CI 1.04 to 3.30) (table 3).

**Table 3:** Logistic regression analysis of risk of all-grade dermatological and endocrine toxicity in patients receiving immune checkpoint inhibitors

	Dermatological toxicity		Endocrine toxicity	
	Univariate	Multivariable	Univariate	Multivariable
**Age category**				
<65 years	1.00 (ref)	1.00 (ref)	1.00 (ref)	1.00 (ref)
65–74 years	1.21 (0.71 to 2.05)	1.34 (0.77 to 2.30)	0.93 (0.55 to 1.56)	1.08 (0.63 to 1.83)
≥75 years	1.69 (0.97 to 2.95)	1.85 (1.04 to 3.30)	0.42 (0.21 to 0.84)	0.43 (0.21 to 0.87)
**Primary site**				
Melanoma	1.00 (ref)	1.00 (ref)	1.00 (ref)	1.00 (ref)
NSCLC	0.62 (0.35 to 1.08)	0.69 (0.39 to 1.23)	0.47 (0.24 to 0.89)	0.42 (0.21 to 0.82)
RCC	0.75 (0.40 to 1.41)	0.95 (0.49 to 1.86)	1.28 (0.70 to 2.33)	1.16 (0.61 to 2.18)
**Treatment type**				
PD-1 inhibitor	1.00 (ref)	1.00 (ref)	1.00 (ref)	1.00 (ref)
Ipilimumab+nivolumab	2.56 (1.01 to 6.49)	2.46 (0.94 to 6.46)	1.07 (0.35 to 3.29)	0.89 (0.28 to 2.85)
Ipilimumab	1.48 (0.78 to 2.8)	1.61 (0.81 to 3.19)	1.22 (0.61 to 2.44)	1.00 (0.48 to 2.09)

OR by logistic regression for all-grade dermatological and endocrine toxicity (95% CIs).

NSCLC, non-small cell lung cancer; RCC, renal cell carcinoma.

When a sensitivity analysis was implemented by excluding those who were treated with ipilimumab-containing regimens, the proportions of patients with all grade and ≥G3 toxicity did not differ significantly between the youngest, middle and oldest age cohorts (table 4). However, a smaller proportion of patients in the ≥75 years cohort discontinued because of toxicity (2.7%) compared with the <65 years cohort (8.6%, p=0.113) despite a similar median number of cycles (table 4). Similar to the overall cohort, there was a non-significant trend toward increased dermatological toxicity in the oldest cohort (28/101 patients, 27.7%) compared with the younger cohorts (25/146 patients, 17.1% and 24/127 patients, 18.9%, p=0.051) (table 4). Conversely, there was a significant decrease in the proportion of patients who developed endocrine toxicity with age with 24.7% of the youngest, vs 10.9% of the oldest cohort affected (p=0.007).

**Table 4:** Cohort characteristics for overall patient cohort and age-determined cohorts in PD-1 inhibitor monotherapy subgroup

	Overall N (%) (n=374)		<65 years N (%) (n=146)		65–74 years N (%) (n=127)		≥75 years N (%) (n=101)		X^2^	
	All grade	≥G3	All grade	≥G3	All grade	≥G3	All grade	≥G3	All grade	≥G3
**All toxicity**	203 (54.3)	35 (9.4)	76 (52.1)	13 (8.9)	74 (58.3)	16 (12.6)	54 (53.5)	6 (5.9)	NS	NS
**Adverse event**										
Dermatitis	77 (20.6)	2 (0.5)	25 (17.1)	0 (0.0)	24 (18.9)	0 (0.0)	28 (27.7)	2 (2.0)	NS	NS
Lower GI	46 (12.3)	9 (2.4)	14 (9.6)	6 (4.1)	18 (14.2)	2 (1.6)	14 (13.9)	1 (1.0)	NS	NS
Endocrine	71 (19.0)	8 (2.1)	36 (24.7)	3 (2.1)	24 (18.9)	4 (3.1)	11 (10.9)	1 (1.0)	**0.007**	NS
Hepatitis	34 (9.1)	5 (1.3)	17 (11.6)	0 (0.0)	12 (9.4)	4 (3.1)	5 (5.0)	1 (1.0)	NS	NS
Rheumatological	15 (4.0)	2 (0.5)	3 (2.1)	0 (0.0)	7 (5.5)	2 (1.6)	5 (5.0)	0 (0.0)	NS	NA
Other	60 (16.0)	12 (3.2)	23 (15.7)	4 (2.7)	26 (20.5)	7 (5.5)	11 (10.9)	1 (1.0)	NS	NS
	**Overall N (%**) (**n=243**)		**<65 years N (%**) (**n=81**)		**65–74 years N (%) (n=88**)		**≥75 years N (%**) (**n=74**)		**X^2^**	
**Median no of cycles**	**7 (1–60**)		**5 (1–60**)		**7 (1–49**)		**8 (1–33**)		**NA**	
**Discontinuation due to toxicity**	14 (5.8)		7 (8.6)		5 (5.7)		2 (2.7)		NS	

A total of 374 patients overall for which toxicity data are known, 243 patients for which continuation/discontinuation data are known.

GI, gastrointestinal; NA, Insufficient patient numbers for analysis; NS, not significant.

**Figure 1:**
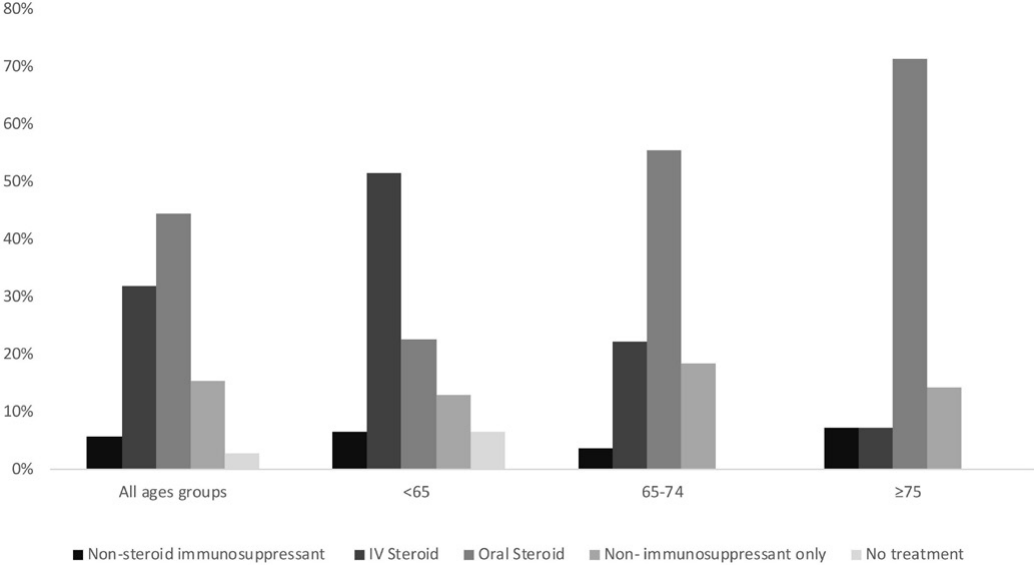
Treatment of ≥G3 toxicities. Patients were categorized into age groups (<65, 65–74 and ≥75 years). For each episode of toxicity, the most potent treatment modality only was recorded (non-steroid immunosuppressant>intravenous steroid>oral steroid>non-immunosuppressant only>no treatment). Non-steroid immunosuppressant treatment included biologics (eg, infliximab) and systemic immunosuppressants (eg, mycophenolate mofetil). Figures are shown as percentage of the total (for all age groups and each age group respectively). Treatment data were available for 302 patients.

In terms of treatment of ≥G3 toxicities oral steroids were the main treatment modality, used in 44.4% of episodes and intravenous steroids were used in 31.9% of episodes (figure 1, online supplementary table S1). Treatment for all ≥G3 toxicities (and GI toxicity) differed between the age groups, with higher intravenous steroid use in the younger age groups (51.6% in <65 years and 22.2% in 65–74 years groups) compared with the ≥75 years group (7.1%), with more of the oldest patients being treated with oral steroids instead.

10.1136/jitc-2020-000871.supp1 Supplementary data

## Discussion

Our study did not observe a significant difference in overall toxicity between age cohorts, although there was a numerical trend toward lower all-grade and ≥G3 toxicities in the ≥75 years compared with the younger cohort, and the discontinuation rates for toxicity were lowest in the oldest age cohort. This result is consistent with findings from previous studies including a meta-analysis of multiple clinical trials.^22^ However, we did observe significantly higher endocrine toxicity in younger patients, and conversely, dermatological toxicity showed the reverse trend, even in the setting of PD-1 inhibitor monotherapy.

Previous studies examining the association of age with toxicity of ICIs have focused on single tumor types or single agents, or have had fewer than 250 patients in total.^17–19 23^ These studies have also used varying cut-offs to classify the oldest cohort and in clinical trials, subgroup analysis is usually performed on patients <or ≥ 65 years old, therefore limiting specific inferences about patients ≥75 years.

Although we found decreased endocrine toxicity in older patients, a previous study specifically looking at patients with melanoma receiving ICI therapy showed the opposite trend, of increasing incidence of autoimmune endocrinopathy with age.^21^ This may reflect disease-specific effects of ICI therapy or other factors inherent to the demographics of the study populations including the fact that many patients in the previous study had received ipilimumab prior to PD-1 inhibition.

As with the prevalence of certain toxicities across different age cohorts, the treatment of these toxicities also showed interesting age-dependent patterns. There was a strong trend toward lower intravenous steroid use in the older cohorts, with more of these patients being treated with oral steroids instead. The exact reasons underlying this difference are unclear. However, it is possible that there is a level of reluctance to utilize intravenous steroids in older patients, due to fear of side effects. Furthermore, there is a spectrum of severity, even within the same grade of toxicity, thus, it is possible that older patients experienced toxicities at the lower end of this spectrum compared with younger patients, thereby requiring less potent treatment. In future studies, outcomes for these patients could be analyzed to elucidate the impact of IV or oral steroid use on the recovery of patients from immunotherapy induced toxicities.

Only treatment type (PD-1/PD-L1 inhibitors vs CTLA4 inhibitors) was significantly associated with all grade toxicity using logistic regression, with the risk of toxicity significantly greater in the cohort receiving ipilimumab-containing therapy. However, patients on ipilimumab-containing therapy were under-represented in our eldest cohort (eight patients), thus making it difficult to infer the real-world toxicity profile of this regimen relative to younger patients. Future studies are required to address this issue.

One of the main strengths of our study is the analysis of real-world data from multiple centers. Furthermore, a substantial proportion of our data came from patients of ages that are under-represented in clinical trials, yet who make up a large proportion of daily practice. Limitations include the retrospective nature of the data collection, the small number of patients on ipilimumab-containing therapies, and the use of age alone to define cohorts. Although age is a risk factor for frailty and decreased physiological reserve, there is a large heterogeneity of functional status in older patients. In future studies, specific measures of frailty and functional status will be necessary to broaden understanding of toxicity in the oldest aged cohorts.

In summary, we have analyzed a large real-world dataset across different tumor and treatment types, examining the effect of age on the toxicity of ICIs. We have shown that, outside clinical trial settings, immunotherapy is tolerated similarly across age groups with no evidence for poorer tolerance in the oldest age groups. The only determinant of toxicity in our study was treatment type, with anti-CTLA-4 containing therapies associated with increased rates of toxicities. In terms of site-specific toxicity, we found that endocrine toxicity was more common in younger patients with the opposite trend for dermatological side effects. More data are needed on the use of CTLA-4 inhibitors in older people, and the impact of frailty and functional status on outcomes, however, PD-1 inhibitors appear to be safe and well tolerated in patients considered suitable to receive treatment.

## Data Availability

All data relevant to the study are included in the article or uploaded as online supplementary information. For further enquiries please contact the corresponding author.
