# Supplementary material for: Impact of age on the toxicity of immune checkpoint inhibition
Source: J Immunother Cancer. 2020 Oct 8;8(2):e000871. doi: 10.1136/jitc-2020-000871 (PMC7545628; doi:10.1136/jitc-2020-000871)
Supplement: Supplementary data [file jitc-2020-000871supp001.pdf]

BMJ Publishing Group Limited (BMJ) disclaims all liability and responsibility arising from any reliance placed on this supplemental material which has been supplied by the author(s)

Supplementary Table

Table S1: Treatment modalities used for ≥G3 toxicities

**Overall Toxicities ≥G3**	**Overall**	**<65 yrs**	**65-74 yrs**	**≥75 yrs**	**Chi Squared**
Total episodes / Treatment	**72**	**31**	**27**	**14**	
Non-steroid immunosuppressant	4 (5.6%)	2 (6.5%)	1 (3.7%)	1 (7.1%)	NA
IV Steroid	23 (31.9%)	16 (51.6%)	6 (22.2%)	1 (7.1%)	
Oral Steroid	32 (44.4%)	7 (22.6%)	15 (55.6%)	10 (71.4%)	
Non- immunosuppressant only	11 (15.3%)	4 (12.9%)	5 (18.5%)	2 (14.3%)	
No treatment	2 (2.8%)	2 (6.5%)	0 (0.0%)	0 (0.0%)	
**Dermatitis**	**Overall**	**<65 yrs**	**65-74 yrs**	**≥75 yrs**	
Total episodes / Treatment	**3**	**0**	**2**	**1**	
IV Steroid	1 (33.3%)	0 (0.0%)	1 (50.0%)	0 (0.0%)	NA
Oral Steroids+ Topical steroid	2 (66.7%)	0 (0.0%)	1 (50.0%)	1 (100.0%)	
**Lower GI**	**Overall**	**<65 yrs**	**65-74 yrs**	**≥75 yrs**	
Total episodes / Treatment	**24**	**13**	**6**	**5**	
Non-steroid immunosuppressant	2 (8.3%)	1 (7.7%)	1 (16.7%)	0 (0.0%)	NA
IV Steroid	13 (54.2%)	11 (84.6%)	2 (33.3%)	0 (0.0%)	
Oral Steroid	9 (37.5%)	1 (7.7%)	3 (50.0%)	5 (100.0%)	
**Endocrine**	**Overall**	**<65 yrs**	**65-74 yrs**	**≥75 yrs**	
Total episodes / Treatment	**11**	**4**	**5**	**2**	
Oral Steroid	5 (45.5%)	2 (50.0%)	2 (40.0%)	1 (50.0%)	NA
Non- immunosuppressant only	6 (54.5%)	2 (50.0%)	3 (60.0%)	1 (50.0%)	
**Hepatitis**	**Overall**	**<65 yrs**	**65-74 yrs**	**≥75 yrs**	
Total episodes / Treatment	**13**	**4**	**5**	**4**	
Non-steroid immunosuppressant	2 (15.4%)	1 (25.0%)	0 (0.0%)	1 (25.0%)	NA
IV Steroid	4 (30.8%)	1 (25.0%)	2 (40.0%)	1 (25.0%)	
Oral Steroid	7 (53.8%)	2 (50.0%)	3 (60.0%)	2 (50.0%)	
**Rheumatological**	**Overall**	**<65 yrs**	**65-74 yrs**	**≥75 yrs**	
Total episodes / Treatment	**4**	**2**	**2**	**0**	
IV Steroid	1 (25.0%)	1 (50.0%)	0 (0.0%)	0 (0.0%)	NA
Oral Steroid	3 (75.0%)	1 (50.0%)	2 (100.0%)	0 (0.0%)	
**Other Toxicities**	**Overall**	**<65 yrs**	**65-74 yrs**	**≥75 yrs**	
Total episodes / Treatment	**17**	**8**	**7**	**2**	
IV Steroid	4 (23.5%)	3 (37.5%)	1 (14.3%)	0 (0.0%)	NA
Oral Steroid	6 (35.3%)	1 (12.5%)	4 (57.1%)	1 (50.0%)	
Other treatment only	5 (29.4%)	2 (25.0%)	2 (28.6%)	1 (50.0%)	
No Treatment	2 (11.8%)	2 (25.0%)	0 (0.0%)	0 (0.0%)	

NA – not enough data for meaningful analysis
